# Evidence for the Beneficial Effects of Brazilian Native Fruits and Their By-Products on Human Intestinal Microbiota and Repercussions on Non-Communicable Chronic Diseases—A Review

**DOI:** 10.3390/foods12183491

**Published:** 2023-09-19

**Authors:** Maiara da Costa Lima, Heloísa Maria Almeida do Nascimento, Jaielison Yandro Pereira da Silva, José Luiz de Brito Alves, Evandro Leite de Souza

**Affiliations:** Department of Nutrition, Health Science Center, Federal University of Paraíba, João Pessoa 58051-900, PB, Brazil; maiaraclima@gmail.com (M.d.C.L.); heloysa28@gmail.com (H.M.A.d.N.); jaielison@gmail.com (J.Y.P.d.S.); jose.luiz@academico.ufpb.br (J.L.d.B.A.)

**Keywords:** fruit, bioactive compounds, functional foods, intestinal microbiome, chronic diseases

## Abstract

Non-communicable chronic diseases (NCDs) are the most widespread cause of mortality worldwide. Intestinal microbiota balance can be altered by changes in the abundance and/or diversity of intestinal microbiota, indicating a role of intestinal microbiota in NCD development. This review discusses the findings of in vitro studies, pre-clinical studies and clinical trials on the effects of Brazilian native fruits, their by-products, as well as their bioactive compounds on human intestinal microbiota and NCD. The major bioactive compounds in Brazilian native fruits and their by-products, and the impacts of their administration on outcomes linked to intestinal microbiota modulation are discussed. Mechanisms of intestinal microbiota affecting NCD could be linked to the modulation of absorption and energy balance, immune and endocrine systems, and inflammatory response. Brazilian native fruits, such as acerola, açaí, baru, buriti, guava, jabuticaba, juçara, and passion fruit, have several bioactive compounds, soluble and insoluble fibers, and a variety of phenolic compounds, which are capable of changing these key mechanisms. Brazilian native fruits and their by-products can help to promote positive intestinal and systemic health benefits by driving alterations in the composition of the human intestinal microbiota, and increasing the production of distinct short-chain fatty acids and phenolic metabolites, thereby enhancing intestinal integrity and homeostasis. Evidence from available literature shows that the modulatory impacts of Brazilian native fruits and their by-products on the composition and metabolic activity of the intestinal microbiota could improve several clinical repercussions associated with NCD, reinforcing the influence of intestinal microbiota in extra-intestinal outcomes.

## 1. Introduction

Non-communicable chronic diseases (NCDs) comprise a group of illnesses, such as hypertension, obesity, dyslipidemias, diabetes, and cancer, recognized as the most widespread cause of mortality worldwide [1]. Behavioral risk factors, including high calorie and saturated fat intake, sedentary lifestyle, tobacco smoking, excessive alcohol consumption, and low fruit and vegetable consumption, have been linked to NCD development [1]. Recent investigations have suggested a relationship between NCD, intestinal microbiota imbalance and intestinal barrier disruption [2,3,4,5,6].

A plant food-rich diet is part of a central dietary intervention for preventing and treating NCD due to its antioxidant and anti-inflammatory properties linked to the presence of several bioactive compounds, nutrients, and low caloric density [7,8,9]. Regular fruit consumption increases intestinal microbiota diversity, composition, and metabolite production [7,10]. Brazil has a vast flora due to the existence of different biomes (Caatinga, Cerrado, Pampa, Atlantic Forest, and Amazon) with specific edaphoclimatic conditions, resulting in the presence of several native fruits with remarkable nutritional composition and a variety of bioactive compounds with reported health-related properties. These plant fruit species have been growing in Brazil for hundreds of years or longer, being present in these territories prior to the introduction of non-native plants from other continents, and they are well adapted to the edaphoclimatic conditions that characterize the distinct Brazilian biomes [11,12,13,14,15].

In addition to the availability of several Brazilian native fruits for in natura consumption, the processing of fruits produces high amounts of by-products, commonly comprising remnants of flesh pulp, peels, and/or seeds, which usually exceed 1/3 of the total fruit weight. These by-products contain a variety of compounds of biotechnological interest, including enzymes, vitamins, and bioactive molecules such as phenolics, alkaloids, flavonoids, carotenoids, glycosides, tannins, saponins, terpenoids, and dietary fibers [14], making them promising matrices for utilization as functional ingredients due to their reported antimicrobial, antioxidant, and prebiotic properties [15,16,17,18].

Especially, phenolic compounds, typically found in high contents in fruits and their by-products, offer several health benefits due to their reported antioxidant and anti-inflammatory effects linked to inhibiting pro-inflammatory cytokines (e.g., tumor necrosis factor-alpha—TNF-α, and interleukins). Phenolic compounds could provide local health benefits by directly interacting with the gastrointestinal tract and intestinal microbiota, preventing the development of NCDs, such as diabetes, obesity, cardiovascular disorders, and cancer [19].

This review presents and discusses the research evidence of in vitro and pre-clinical studies and clinical trials regarding the effects of Brazilian native fruits, their by-products, as well as their bioactive compounds on human intestinal microbiota and the reported related repercussions in NCD. 

## 2. The Potential Role of Intestinal Microbiota in NCD

NCDs are a group of diseases with no transmissible or noninfectious etiology, including obesity, cardiovascular diseases, diabetes mellitus, and cancer [1], whose occurrence are linked to genetic, environmental, behavioral, and even social factors [6]. An inadequate diet pattern is an important etiologic factor increasing NCD risk, especially high-sugar, high-saturated fat, high energy, and low-fiber consumption [20,21,22,23,24,25].

Obesity is characterized by excessive accumulation or abnormal body fat distribution, affecting health and well-being [26]. Excessive adipose tissue accumulation produces proinflammatory cytokines, causing low-grade chronic inflammation, oxidative stress, insulin resistance, and endothelial dysfunction [27]. Individuals with obesity have a higher risk of developing endothelial dysfunction, commonly linked to atherosclerosis development and progression [28,29].

Dyslipidemia consists of alterations in lipid metabolism with an increase in low-density lipoprotein cholesterol (LDL-c), triglycerides, total cholesterol, and/or low high-density lipoprotein cholesterol (HDL-c) levels. It is a recognized risk factor for atherogenic disease [30]. The endothelial dysfunction increases the permeability of the vascular endothelium and produces intimate tunic LDL-c infiltration. Circulating monocytes are recruited in the intimate tunic and transformed into macrophages, producing proinflammatory cytokines, injury to endothelium, platelet adhesion, inflammatory activity, and activation in dysfunctional areas [31], increasing the risk of thrombosis and stroke [32].

Diabetes mellitus (DM) is characterized by persistent hyperglycemia caused by insulin resistance and/or impairment in insulin production by pancreatic beta cells [33]. Chronic hyperglycemia produces metabolic alterations and affects different organs or physiological processes. The endothelial cells can be damaged, reducing the ability of the tissue to provide protection against oxidative and inflammatory injury. The high glucose serum level increases endothelium infiltration and improves plaque remodeling [34].

**Figure 1:**
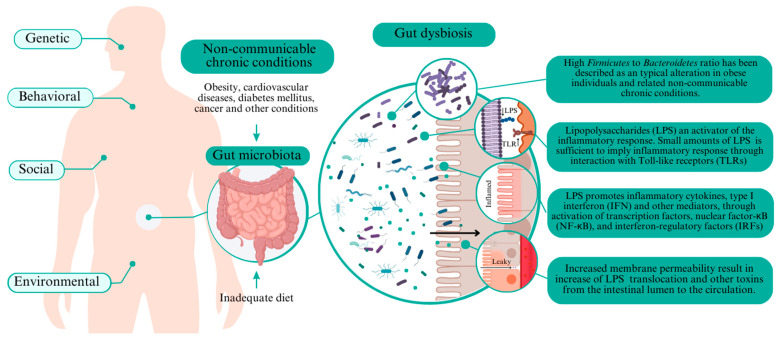
Key mechanisms of dysbiosis affecting non-communicable chronic diseases.

Cancer is a critical NCD, especially colorectal cancer, which is one of the most prevalent cancer types with almost two million cases reported yearly [23,35]. Chronic inflammation promotes gene transcription that improves metaplasia and cancer development, including colorectal cancer [23]. Figure 1 summarizes the primary mechanisms associated with intestinal dysbiosis and NCD.

Nutrition management is part of the clinical care for individuals with NCD where regular consumption of vegetables, including whole grains, fruits, green leafy vegetables, and tea, has been highly recommended as a preventive strategy [20]. Fruit-rich diets could induce beneficial intestinal microbiota modulation due to the increased ingestion of several bioactive constituents, including fibers and phenolic compounds [36,37].

Intestinal microbiota derangements, including decreased richness and diversity and increased gut barrier permeability, are linked to host–microbiome impairment and increased risk for NCD development [3,5,6]. The intestinal microbiota is described as a vast diversity of microorganisms within a symbiotic interaction [38], with the ability to digest nutrients to provide energy and vitamins, modulate host immune reactions, inhibit pathogens, produce neurotransmitters, influence host-cell proliferation, and regulate intestinal endocrine functions [39,40]. Alterations in the abundance and/or diversity of intestinal microbiota could alter the intestinal microbiome equilibrium and lead to intestinal and extra-intestinal diseases [5,6].

The specific intestinal microbiota profile putatively associated with NCD development is still not understood since the available evidence has differences in methodological aspects, including the population evaluated, geographical localization, diet, age, gender, genetic, and distinct environmental factors affecting the intestinal microbiota [41,42,43,44].

A high *Firmicutes* to *Bacteroidetes* ratio in intestinal microbiota is a typical alteration in individuals with obesity and related diseases [44,45], since *Firmicutes* could be more effective in extracting energy, especially from carbohydrates, thereby affecting the energetic metabolism [46]. Increases in Proteobacteria abundance in the intestine have been commonly found in individuals with obesity [46,47,48]. Proteobacteria is among the most abundant intestinal microbiota phyla and includes various pathogenic microorganisms (e.g., Enterobacteriaceae members). One similarity of these bacterial groups is their Gram-negative staining and the presence of lipopolysaccharides (LPS) in the outer membrane [49].

LPS are endotoxins that activate an inflammatory response. Small LPS amounts in the blood should trigger an inflammatory response via interaction with Toll-like receptors (TLR) [50]. TLRs are essential in the innate immune system to detect microbial infection. Immune response by LPS induces the release of inflammatory cytokines, type I interferon (IFN), and other mediators through the activation of transcription factors, nuclear factor-κB (NF-κB), and interferon-regulatory factors (IRFs) [50]. Chronic immune responses induced by LPS lead to low-grade systemic inflammation and trigger chronic disease pathways [29,51].

Proinflammatory cytokines (e.g., IFNγ) increase the tight junction permeability due to a decreased ZO-1 (Zonula occludens-1) protein expression, thereby suppressing the tight junction barrier function [50]. The increased membrane permeability in intestinal dysbiosis causes increased translocation of LPS and other toxins from the intestinal lumen to the circulation, achieving bloodstream and potentially extra-intestinal cellular targets [6,29].

The receptor for advanced glycation end products (RAGE) is a transmembrane glycoprotein expressed in most organs and could bind several compounds (multiligand receptor). RAGE signaling drives primary inflammatory mechanisms in several cells linked to the development and progression of pathological conditions, including diabetes, cardiovascular diseases, and cancer [6,52]. RAGE can directly bind LPS and activate proinflammatory signaling independent of the primary receptor for LPS (i.e., Toll-like receptor 4—TLR4) [52].

A high-fat diet (HFD) used to induce several diseases in animal models, such as stress oxidation, dyslipidemia, obesity, hyperinsulinemia, hyperglycemia, and hypertension [15,53,54,55,56], produces impairments in intestinal microbiota, including decreased α-diversity, increased abundance of *Firmicute* and *Proteobacteria* species, and reduced abundance of *Bacteroides*, *Actinobacteria*, *Akkermansia muciniphila*, *Parabacteroides distasonis*, and *Bacteroides acidifaciens*. HFD also increases LPS plasma concentration and decreases claudin protein expression [29,54,56]. Claudin is a transmembrane protein in intestinal epithelia that composes the strands in tight junction plaques in the intercellular space, with a role in intestinal permeability alteration [57].

A previous investigation showed that mice fed an HFD had increased fasting serum glucose levels and insulin resistance, besides the increased abundance of *Firmicutes* and *Proteobacteria* and the decreased abundance of *Bacteroidetes* and *Verrucomicrobia* in intestinal microbiota [54]. *Firmicutes*, *Clostridia*, and *Negativicutes* were the predominant phyla, and *Verrucomicrobia*, *Bacteroidetes*, *Proteobacteria*, and *Elusimicrobia* phyla were the less abundant phyla in individuals with obesity and DM type 2 (DM2) [58]. Another study showed that individuals with obesity and DM2 had a lower abundance of *Bifidobacterium*, Bacteroidetes, and Firmicutes than the non-diabetic group [59]. Intestinal microbiota alterations in individuals with DM2 could also induce low-grade inflammation and insulin resistance [59].

The role of the intestinal microbiota in colorectal cancer is reported [5,60,61]. Alterations in the intestinal microbiota community, including the presence of *Fusobacterium nucleatum* [62,63], *Escherichia coli* psk+ [64,65], and *Clostridium* species, could have an important role in colon cancer development and progression [5]. Intestinal microbiota may produce carcinogenesis by genotoxicity, such as through colibactin production, activation of Toll-like receptor 2 (TLR2)/TLR4 signaling and inhibition of apoptosis, suppression of host immunity response, and/or production of metabolites (e.g., lactic acid) that increase cancer progression [5,60,62,64].

Probiotics are “live microorganisms that, when administered in adequate amounts, confer a health benefit to the host” [66,67]. These microorganisms could be found in intestinal human microbiota in symbiosis with the host [28], and have been continually used to improve microbiota homeostasis and support human intestinal health [68].

Species of *Bifidobacterium*, *Streptococcus*, and *Lactobacillus*, including some of the recently reclassified genera, are traditionally used as probiotics due to their typical safety and biological properties [16,44,69], which are strain-specific features [19]. The most frequently reported beneficial effects of probiotic consumption include immune system modulation, organic acid production, and defense against intestinal pathogens [68,70,71,72]. However, recent investigations have suggested the role of probiotics against several NCD, such as DM2 [73,74], dyslipidemia, atherosclerosis, hypertension, and cardiovascular disease [75,76,77], by reducing chronic inflammation and improving intestinal microbiota composition and metabolism [19,75,77].

Food bioactive compounds could modulate the intestinal microbiota by primarily stimulating beneficial bacterial groups and increasing the production of bioactive metabolites, including short-chain fatty acids (SCFAs) (e.g., acetic, propionic, and butyric acids) [15,78,79,80,81,82]. In the intestinal environment, SCFA produced by healthy intestinal microbiota, which is induced by a fruit-rich diet, could cause several beneficial effects on intestinal and extra-intestinal targets [39]. SCFAs are metabolites produced from microbial fermentation of non-digested or partly digested substrates, including polysaccharides [83]. Different tissues, such as the intestine, adipose tissue, skeletal muscle, and immune cells, express SCFA-sensing G-protein-coupled receptors (GPCRs), causing several physiological reactions [83,84]. In enteroendocrine cells, SCFA bind G protein-coupled receptor (GPCR) to produce glucagon-like peptide 1 (GLP-1) and peptide YY (PYY) [39], which via vagal activation stimulate insulin secretion, decrease gastric emptying, increase energy oxidation, and inhibit food intake [39,85].

SCFAs regulate the differentiation, activation, and apoptosis of dendritic, macrophage, and T cells, besides reducing proinflammatory cytokines and expression of tumor necrosis factor (TNF) and nitric oxide synthase (NOS) in monocyte, contributing to intestinal homeostasis [84]. In the blood–brain barrier (BBB), i.e., hemato-encephalic barrier, SCFAs improve integrity and reduce permeability to toxic molecules, playing a role in the central nervous system (CNS) homeostasis [6,86].

## 3. Bioactive Compounds in Brazilian Native Fruits

Regular consumption of fruits and other plant-origin foods rich in vitamins, minerals, fibers, and phenolic compounds has several health benefits. Brazilian native fruits include a vast diversity of edible species [13]. Mainly, acerola (*Malpighia emarginata* D.C.), açaí (*Euterpe oleracea* Mart.), baru (*Dipteryx alata* Vog.), buriti (*Mauritia flexuosa* L.), guava (*Psidium guajava* L.), jabuticaba (*Myrciaria jaboticaba* (Vell.) Berg), juçara (*Euterpe edulis* Mart.), and passion fruit (*Passiflora capsularis* L.) are widely cultivated and available in Brazil; they are reported as sources of bioactive components that are capable of modulating the composition and metabolic activity of the intestinal microbiota with local and systemic benefits, including anti-inflammatory and antioxidant activity, better insulin sensitivity, body weight reduction, and effective management of dyslipidemia, besides having evidence of low genotoxic [10,15,33,44,48,54,81,87]. Table 1 summarizes the major bioactive compounds found in peel, seed, and/or pulp of Brazilian native fruits that are being investigated for their ability to modulate intestinal microbiota.

Fruits are important dietary fiber sources, characterized as edible carbohydrate polymers (three or more monomeric units) resistant to the action of digestive intestinal enzymes [15,94,95]. Soluble fibers, i.e., pectin, mucilage, and gum, can hold water and develop gel in the gastrointestinal tract. This leads to the bonding of ions and bile acids, thereby reducing the absorption of lipids and simple carbohydrates. The gel-forming ability of dietary fiber increases fecal bulk through stimulation of the intestinal microbiota and improves cholesterol, lipid, and glucose metabolism and gastric emptying [4]. Insoluble fibers, i.e., cellulose, hemicellulose, and lignin, increase intestinal motility and fullness, helping to reduce food intake [94].

Brazilian native fruits typically have high contents of phenolic compounds and presence of anthocyanidins [10,12,54,96,97]. Phenolic compounds include a wide diversity of bioactive molecules with an aromatic ring bond to a hydroxyl group, and have important anti-inflammatory, antimicrobial, and antioxidant properties [6,18,98,99,100,101,102]. In addition, phenolic compounds may exert prebiotic activity because of their metabolization by the intestinal microbiota with selective stimulatory effects on beneficial bacterial populations and can thereby remodel the metabolic activity of the intestinal microbiota [16,17,82,87,95,103].

Due to their richness in nutrients and bioactive compounds, Brazilian native fruits are important food choices for preventing and alleviating the symptoms of several NCD [10,103,104,105,106,107]. Table 2 shows some experimental details (type of study, intestinal microbiota condition, fruit evaluated, and dose) of retrieved studies evaluating the impacts of Brazilian native fruits and their by-products on human intestinal microbiota. Next, the effects of Brazilian native fruits and their by-products on the intestinal microbiota and the reported primary clinical outcomes linked to NCDs in these in vitro, preclinical, and clinical studies are presented and discussed.

## 4. Potential Effects of Brazilian Native Fruits and Their By-Products on Intestinal Microbiota and Clinical Outcomes

Table 3 summarizes the main results of the retrieved studies measuring the effects of Brazilian native fruits and their by-products on intestinal microbiota and reported clinical repercussions.

### 4.1. Acerola (Malpighia emarginata D.C.)

Acerola is a popular tropical fruit native to America and cultivated in Brazil, mainly in the northeast [9,16]. Acerola by-product, either subjected or not subjected to simulated gastrointestinal digestion (GID) (20%, *w*/*v*), supported the growth of probiotic *Lactobacillus acidophilus* LA-05, *Lacticaseibacillus casei* L-26, and *Bifidobacteruim animalis* subsp. *lactis* BB-12 in laboratory medium. During 48 h of fermentation, acerola by-product increased the viable cell counts of probiotics strains (>9 log UFC/mL). Cultivation of probiotics with acerola by-product results in carbohydrate consumption, thereby decreasing the pH and increasing the production of organic acids over time, namely citric, succinic, lactic, acetic, formic, and malic acids [16]. The digested acerola by-product was subjected to an in vitro colonic fermentation using a pooled fecal inoculum from healthy subjects (25- to 40 years old) for 24 h. The changes in the relative abundance of target intestinal bacterial groups were measured using fluorescence in situ hybridization (FISH) coupled with flow cytometry (FC) [16]. The in vitro colonic fermentation with acerola by-product increased the relative abundance of *Bifidobacterium* spp. (4.87%; negative control: 2.3%), *Lactobacillus* spp./Enterococcus spp. (3.90%; negative control: 3.2%), *Bacteroides* spp./*Prevotella* spp. (4.80%, negative control: 4.6%), and *Eubacterium rectale*/*Clostridium coccoides* (6.0%, negative control: 4.3%), as well as the production of butyric (from 1.25 to 1.64 g/L), propionic (from 0.82 to 0.93 g/L), and acetic acid (from 0.75 to 1.33 g/L), indicating that acerola by-product could selectively modulate bacterial groups forming the intestinal microbiota and increase the production of health-related metabolites [16].

**Table 3:** Studies assessing the effects of native Brazilian fruit in intestinal microbiota and reported clinical repercussions (when applicable).

In Vitro Studies			
Brazilian Fruit	Bioactive Compounds	Main Results	Reference
Acerola (*Malpighia emarginata* D.C.)	Insoluble and soluble dietary fibers, cyanidin3-rhamnoside, (+)-catechin, isorhamnetin, 2,5-dihydroxybenzoic acid, catechin, myricetin, salicylic acid, and rutin.	Acerola and guava by-products previously submitted to simulated gastrointestinal digestion increased the abundance of *Bifidobacterium* spp., *Lactobacillus* spp./*Enterococcus* spp., *Bacteroides/Prevotella*, and *E. rectale*/*C. coccoides*, and decreased the abundance of *C. histolyticum* during colonic fermentation, besides increasing SCFA concentration and decreasing pH.	[15,16]
Acerola (*Malpighia emarginata* D.C.)	Insoluble and soluble dietary fibers, cyanidin3-rhamnoside, (+)-catechin, isorhamnetin, 2,5-dihydroxybenzoic acid, catechin, myricetin, salicylic acid, and rutin.		
Guava (*Psidium guajava* L.)	Insoluble and soluble dietary fibers, 3,4 dihydroxybenzoic acid, salicylic acid, 2,5-dihidorxybenzoic acid, myricetin, synapic acid.		
Açaí (*Euterpe oleracea* Mart.)	Gallic acid, protocatechuic acid, *p*-hydroxybenzoic acid, gentisic acid, chlorogenic acid, caffeic acid, syringic acid, ferulic acid, trains-cinnamic acid, quercetin, vanillic acid, cyanidin-3-*O*-glucoside, cyanidin-3-*O*-rutinoside, pelargonidin-3-*O*-glucoside, and peonidin-3-*O*-rutinoside.	Açaí pulp digestion resulted in the degradation of phenolic compounds, decreased abundance of *Bacteroides*/*Prevotella* spp. and *Clostridium-histolyticum* groups, and increased SCFA. Açaí caused no cytotoxic effects in HT29 cells and reduced DNA damage, indicating an anti-genotoxicity effect.	[87]
Buriti (*Mauritia flexuosa* L.)	Insoluble and soluble fibers, isoquercetin, ferulic acid, vanillic acid, caffeic acid, and quercetin.	Fermented milk with passion and buriti fruit into SHIME^®^ resulted in an increased abundance of *Bacteroidetes* and *Actinobacteria*, reduced abundance of Proteobacteria phyla, decreased ammonia amounts, and increased the contents of propionic and butyric acids.	[48,88]
Passion (*Passiflora capsularis* L.)	Insoluble and soluble fibers, phenolic compounds, and carotenoids.	Low-fat goat milk fermented by *Lactobacillus casei* Lc-1 and supplemented with passion fruit by-product decreased the abundance of *Prevotella*, *Megamonas*, and *Succinivibrio* genera, and increased the abundance of *Lactobacillus* and *Bifidobacterium* genera.	[44]
Jabuticaba (*Myrciaria jaboticaba* (Vell.) Berg)	Castalagin, vescalagin, procyanidin a2, ellagic acid, gallic acid, cyanidin-3-*O*-glucoside, delphinidin-3-*O*-glucoside, malvidin-3-*O*-glucoside, pelargonidin-3-*O*-glucoside, peonidin, and hesperidin.	Simulated gastrointestinal digestion and colonic fermentation of jabuticaba by-product increased the contents of caftaric acid, gallic acid, catechin, epicatechin gallate, procyanidin A1, procyanidin B1, and procyanidin B2, and decreased the contents of cyanidin 3-glucoside, delphinidin 3-glucoside, epicatechin, hesperidin, kaempferol 3-glucoside, and cis-resveratrol. Jabuticaba by-product decreased pH, increased SCFA, increased the population of *Lactobacillus* spp./*Enterococcus* spp., *Bifidobacterium* spp., and *E. rectale*/*C. coccoides*, and decreased the population of *Bacteroides* spp./*Prevotella* spp. during colonic fermentation.	[17,55]
		Consumption of the yogurt supplemented with lyophilized jabuticaba seed extract increased alpha diversity and equalized the bacterial biodiversity in feces, as well as promoted cytotoxic effects on cancer cells and exhibited antioxidant activity by reducing reactive oxygen species generation.	[55,90]
Juçara (*Euterpe edulis* Mart.)	Cyanidin-3-rutinoside, cyanidin-3-glucoside, malvidin-3-glucoside, peonidin-3-rutinoside, pelargonidin-3-glucoside, rutin, quercetin, and p-coumaric acid.	Juçara pulp colonic fermentation increased the abundance of *Bifidobacterium* spp., *Eubacterium rectale*/*Clostridium coccoides* group and *Bacteroides* spp./*Prevotella*, reduced the abundance of *Clostridium histolyticum*, and caused no alteration in populations of *Lactobacillus/Enterococcus* spp.	[91]
Preclinical studies			
Acerola (*Malpighia emarginata* D.C.)	Insoluble and soluble dietary fibers, cyanidin3-rhamnoside, (+)-catechin, isorhamnetin, 2,5-dihydroxybenzoic acid, catechin, myricetin, salicylic acid, and rutin.	Consumption of guava and acerola by-products improved lipid metabolism and weight loss, decreased fecal pH, increased fat excretion, decreased liver fat accumulation, maintained the integrity of crypts, goblet cells, and epithelial cells at the colon, decreased the fecal viable cell counts of Enterobacteriaceae and increased *Bifidobacterium* spp. and *Lactobacillus* spp. viable cell counts. Acerola by-product improved fecal moisture and total SCFA contents in the feces.	[15,16]
Guava (*Psidium guajava* L.)	Insoluble and soluble dietary fibers, 3,4 dihydroxybenzoic acid, salicylic acid, 2,5-dihidorxybenzoic acid, myricetin, and synapic acid.		
Açaí	Cyanidin-3-rutinoside, cyanidin-3-glucoside, and delphinidin-3-glucoside.	Administration of anthocyanin-rich extract of açaí to C57BL/6J obese high-fat-fed mice reduced body weight and lipid accumulation in adipose tissue, improved lipid metabolism, reduced liver damage and steatosis, improved liver function, glucose intolerance, glucose and insulin levels, HOMA-IR index, and reduced lipogenesis-related genes expression. It changed OTU abundance clustering of gut microbiota, decreased the abundance of *Firmicutes* and *Proteobacteria*, and increased the abundance of *Verrucomicrobia* and *Akkermansia muciniphila* in the feces.	[54]
Guava (*Psidium guajava* L.)	Galic acid, epicatchin, cathechin, caffeic acid, kaempferol, chorogenic acid, quercetin, guavinosides A and B, guavinoside C, and psiguadials A and B.	Administration of guava leaves extract improved glucose tolerance and insulin sensitivity and reduced inflammatory cell infiltration, fibrosis in the kidney, fatty liver accumulation, total cholesterol concentration in plasma, expression of hepatic gluconeogenesis-related genes. It increased microbial diversity and species number, *Bacteroidetes* and *Verrucomicrobia* phylum abundance, and decreased *Firmicutes*, *Actinobacteria*, and *Firmicutes* to *Bacteroidetes* ratio in db/db mice.	[33,109]
	Uronic acid, galacturonic acid, galactose, and arabinose.	Administration of guava polysaccharides in C57BL/6 mice reduced weight gain and energy intake, restored lipid metabolism, reduced blood glucose and glucose intolerance, improved liver function, reduced hepatic steatosis and TNF-α level, restored AMPKα phosphorylation, prevented abnormal lipogenesis, and decreased adipose tissue inflammation. It decreased *Firmicutes* to *Bacteroidetes* ratio and abundance of *Mucispirillum* genus, and restored the total SCFA concentration in the feces.	[56]
	Galic acid, epicatchin, cathechin, caffeic acid, kaempferol, chorogenic acid, quercetin, guavinosides A and B, guavinoside C, and psiguadials A and B.	KM mice with induced diarrhea treated with guava extracts reduced stools and diarrhea rate, diarrhea index, and intestinal propulsion rate, increased the α-diversity of the intestinal microbiota, decreased *Deferribacteraceae* family, changed OTU abundance cluster, and restored microbial Alpha diversity in the feces.	[108,109]
Jabuticaba (*Myrciaria jaboticaba* (Vell.) Berg)	Cyanidin-3-*O*-glucoside, delphinidin-3-*O*-glucoside, gallic acid, rutin, myricetin, and quercetin.	Administration of aqueous extract of jabuticaba peel treatment in colitis reduced body weight loss, improved stool consistency score, and spleen enlargement. It increased the viable counts of *Bifidobacteria* spp. and *Lactobacillus* spp., reduced the viable counts of enterobacteria, restored total bacterial aerobic count, and increased butyric and acetic acid production in the feces.	[79]
	Proanthocyanidins, ellagitannins, castalagin, vescalagin, procyanidin A2, and ellagic acid.	Lyophilized jabuticaba seed extract protected against DNA damage, inhibited LDL oxidation, and enhanced the effect of cisplatin (chemotherapy), thereby reducing chromosomal aberrations in A549 cells. Wistar rats receiving yogurt supplemented with jabuticaba extract had decreased aberrant crypt foci in the colon, was protected against colon pathological remodeling, had reduced leukocyte infiltration of the epithelium and lamina propria, COX-2 and TNF-α expression in the colon, and expression of RNA from antiapoptotic cytokines. The seed extract increased bacterial abundance in the feces and restored phylum abundance.	[106]
	Gallic acid, cyanidin-3-*O*-glucoside, delphinidin- 3-*O*-glucoside, malvidin-3-*O*-glucoside, pelargonidin-3-*O*-glucoside, and peonidin.	Freeze-dried jabuticaba peel supplementation reduced weight gain and inflammatory markers expression, and increased cholesterol excretion, SCFA contents, and the abundance of *Bacteroidota* and *Patescibacteria* phylum, besides reducing the relative abundance of *Actinobacteriota* and *Firmicutes* in the feces of black mice (C57BL/6).	[55]
	Ellagic acid, gallic acid, 3,4-dihydroxybenzoic acid, quercetin-3-*O*-rutinoside, myricetin-3-*O*-rhamnoside, quercetin, cyanidin-3-*O*-glucoside, and delphinidin-3-*O*-glucoside.	Freeze-dried jabuticaba peel and seed to C57BL/6 J mice with NAFLD reduced weight gain, glucose intolerance, glucose levels, insulin levels, HOMA-IR index, and total cholesterol, LDL-c level, IL-6 and TNF-α levels, improved liver function, reduced steatosis and fibrosis, improved gut barrier (mucin and claudin), and reduced LPS plasma concentration. It reduced *Firmicutes* to *Bacteroidetes* ratio and the abundance of *Bifidobacteriaceae*, *Mogibacteriaceae*, *Christensenellaceae*, *Clostridiaceae*, *Dehalobacteriaceae*, *Peptococcaceae*, *Peptostreptococcaceae*, and *Ruminococcaceae* in the feces.	[29]
Clinical trial			
Juçara (*Euterpe edulis* Mart.)	Anthocyanins, cyanidin 3-rutinoside, and cyanidin 3-glucoside.	Ingestion of freeze-dried juçara for 6 weeks enhanced fecal acetic acid content and the relative abundance of *A. muciniphila*, *Bifidobacterium* spp., and *C. coccoides.*	[10]

COX-2: ciclo-oxygenase-2, IRS-1: insulin receptor substrate-1, IL-6: interleukin 6, HOMA-IR: Homeostatic Model Assessment for Insulin Resistance, LDL-c: low-density lipoprotein cholesterol, LPS: lipopolysaccharides, KM: Kunming mice, NAFLD: Non-alcoholic fatty liver disease, SCFA: short-chain fatty acids, SHIME^®^: simulator of the human intestinal microbial ecosystem, TNF-α: tumor necrosis factor alfa, OUT: operational taxonomic unit.

Another study examined the viability of *L. paracasei* L-431, *L. acidophilus* LA-5, and *Bifidobacterium longum* BB-46 in acerola by-product during GID. Acerola by-product increased the survival of *L. paracasei* L-431 and *B. longum* BB-46 during GID. Fermentation of acerola by-product with *B. longum* BB-46 by intestinal microbiota of healthy adults in simulator of human intestinal microbial ecosystem (SHIME^®^) confirmed that acerola by-product with *B. longum* BB-46 could modulate the human microbiota composition by decreasing *Clostridium* spp. (typically described as a hostile microorganism to intestinal microbiota) in ascending (vessel 3), transverse (vessel 4), and descending (vessel 5) colon, [87,110], besides decreasing ammonium ion (NH_4_^+^) production in ascending colon (vessel 3) and increasing SCFA production [107].

A pre-clinical investigation showed that acerola by-product (400 mg/kg for 28 days) improved intestinal health and lipid metabolism of Wistar rats with diet-induced dyslipidemia. Administration of acerola by-product improved body weight, decreased visceral fat, VLDL, LDL, and total cholesterol, and increased HDL levels. Acerola by-product improved fecal moisture, fecal fat excretion, and contents of organic acids, as well as reduced the pH of feces. Intestinal microbiota modulation resulted in decreased viable cell counts of Enterobacteriaceae and increased viable cell counts of *Bifidobacterium* spp. and *Lactobacillus* spp., indicating beneficial effects of the phenolic compounds and fibers from acerola by-products in improving intestinal microbiota in dyslipidemia [15].

Acerola by-product has high contents of insoluble (34.35 ± 0.29 g/100 g) and soluble dietary fiber, as well as a variety of phenolic compounds including 2,5-dihydroxybenzoic acid, catechin, myricetin, salicylic acid, and rutin, which could be associated with stimulating effects on probiotics and beneficial intestinal bacterial populations [15]. The phenolic compounds cyanidin3-rhamnoside, (+)-catechin, and isorhamnetin were also found in acerola pulp [9].

### 4.2. Açaí (Euterpe oleracea Mart.)

The colonic fermentation of açaí pulp, a native fruit from the Amazonian region rich in anthocyanins, after a simulated GID reduced the relative abundance of *C. histolyticum* (−0.19 ± 0.10 and −0.24± 0.07 log, respectively) and *Bacteroides* spp./*Prevotella* spp. (−0.14 ± 0.11 and 0.09 ± 0.07 log, respectively) after 8 and 24 h [87]. Açai pulp colonic fermentation increased the concentrations of acetic, propionic, and butyric acids over time. Phenolic compounds in açaí pulp included p-hydroxybenzoic, gentisic, chlorogenic, caffeic, and syringic acid. After the simulated GID and colonic fermentation, quercetin, vanillin, and hydroxybenzoic, chlorogenic, and ferulic acids were detected in acaí pulp, but with a decrease of approximately 50% in their total concentration, suggesting a degradation during digestion and colonic fermentation. In addition, the reported anti-genotoxicity effect (comet assay) showed that açaí pulp reduced DNA damage by about 31.5% [87]. The protective effect on DNA was putatively associated with the antioxidant effects of phenolic compounds found in açaí pulp.

A pre-clinical study showed that açaí anthocyanin-rich extract (150 mg/Kg for 98 days) was effective in decreasing body weight, serum triglycerides, non-esterified fatty acid (NEFA), total cholesterol, and LDL-c levels in C57BL/6J obese mice induced by a high-fat diet. Açaí anthocyanin-rich extract supplementation reduced hepatocyte lipid accumulation, insulin resistance, and modulated intestinal microbiota composition, as indicated by decreased relative abundance of *Firmicutes* and *Proteobacteria* and increased relative abundance of *Verrucomicrobia*, which were alterations markedly induced by an HFD. Açaí extract caused enrichment in *Verrucomicrobia* at the phylum level, *Akkermansia* and *Sporosarcina* at the genus level, and *A. muciniphila* at the species level. *A. muciniphila* abundance negatively correlated with serum triglycerides (TG), glucose, and insulin levels. *Parabacteroides distasonis* and *Bacteroides acidifaciens* negatively correlated with total cholesterol and LDL-c levels. Additionally, *Parabacteroides merdae* negatively correlated with LDL-c levels [54]. A high *A. muciniphila* abundance could putatively prevent obesity and other metabolic disorders via modulating metabolism and energy homeostasis and enhancing insulin sensitivity and glucose hemostasis [111,112]. Results from a double-blind, randomized controlled trial revealed that overweight dyslipidemic individuals consuming açaí pulp (200 g/day for 60 days) in a hyporenergetic diet reduced plasma 8-isoprostane, IL-6, and IFN-γ levels after the intervention, being indicative of reduced oxidative stress [113].

### 4.3. Baru (Mauritia flexuosa L.)

Baru is a fruit from the Cerrado biome, and its commercial fraction, i.e., the baru nut, comprises only 4% of fruit weight, while peel and pulp are considered as the by-products. A previous study investigated the potential prebiotic effects of baru pulp. Fermentation of freeze-dried baru pulp by *L. casei* L-26 resulted in viable cell counts like those in a cultivation medium with glucose or fructooligosaccharides (FOS), a standard prebiotic. The viable cell counts of *B. lactis* BB-12 and *L. acidophilus* LA-05 were higher in medium with baru pulp when compared with medium glucose or FOS, suggesting that baru pulp could be a suitable carbon source for probiotic strains. These results were linked with the reduction in pH, fructose, glucose, and maltose contents, and the production of acetic, propionic, and butyric acids in cultivation media over time. In addition, baru pulp produced a positive prebiotic activity score for *L. casei* L-26 (0.24 ± 0.06), *L. acidophilus* LA-05 (0.18 ± 0.02), and *B. lactis* BB-12 (0.12 ± 0.02). These results indicated that probiotic strains can selectively ferment baru pulp [81]. After 48 h of in vitro fermentation with fecal inoculum from healthy subjects (aged 18–69 years old), baru pulp increased the relative abundance of *Lactobacillus* spp./*Enterococcus* spp., *Bifidobacterium* spp., and *Bacteroides* spp./*Prevotella* spp., and decreased the relative abundance of *E. rectale*/*C. coccoides* and *C. histolyticum*. It indicated a positive effect of the baru pulp on supporting the growth of beneficial bacterial groups forming the human intestinal microbiota [81]. The same study reported high contents of insoluble and various phenolic compounds, including hesperidin, procyanidin B2, epicatechin gallate, chlorogenic acid, and catechin, which were linked to the effects of baru pulp on the intestinal microbiota during colonic fermentation [81].

The high contents of insoluble fibers found in baru pulp [81] can be hydrolyzed by some bacteria forming the intestinal microbiota, such as the *Bacteroides* species [114,115], and they can increase propionic acid production [81,115]. Increased intestinal propionic acid production is associated with decreased systemic inflammation, improved energy balance, and reduced hypertensive cardiovascular damage in mice [116,117].

A pre-clinical study showed that baru nut oil administration (7.2 or 14.4 mL/kg/day for 10 days) reduced thrombus (carotid artery) induced by FeCl_3_ in Wistar rats, besides reducing platelet aggregation, production of the superoxide anion radical in platelets, and improving vascular function [118]. In obese women (age 40 ± 11 years, body mass index 33.3 ± 4.3 kg/m^2^), baru nuts consumption (20 g for 8 weeks) increased glutathione peroxidase (GPx), suggesting a role of the bioactive compounds of baru in improving antioxidant activity [119].

### 4.4. Buriti (Mauritia flexuosa L.)

Buriti pulp is a Brazilian fruit largely consumed in Pantanal, Cerrado, and Amazon Brazilian regions, especially as crude buriti pulp oil. The effects of fermented milk with *L. casei* SJRP38, *Lactiplantibacillus plantarum* ST8Sh, and *Streptococcus thermophilus* TA 080 supplemented with buriti pulp (1%) in the intestinal microbiota of healthy young adults (18 to 20 years old) were evaluated. Milk was administered for 5 days to be fermented in the SHIME^®^ reactor, and the microbial composition was assessed using 16S rRNA sequencing. Fermented milk supplemented with buriti pulp showed a high viable cell count of *L. casei* (8.69 Log CFU/mL) and *L. plantarum* (9.56 Log CFU/mL). Microbiota modulation resulted in an increased relative abundance of *Phocaeicola* and *Firmicutes*, decreased relative abundance of Proteobacteria phyla, and increased relative abundance of Clostridiaceae family and *Alistipes* genus. Clostridiaceae family could play a role in providing energy for colonocytes and protecting the epithelial barrier. Furthermore, acetic, propionic, and butyric acid contents were higher in fermented milk supplemented with the buriti pulp, suggesting it as a substrate that supports probiotic growth, besides modulating the intestinal microbiota and SCFA production [48]. Fresh and freeze-dried buriti pulp and endocarp are sources of insoluble and soluble dietary fiber, including soluble pectin, as well as phenolic compounds, such as isoquercetin, ferrulic acid, vanillic acid, caffeic acid, and quercetin, which could account for the reported effects on intestinal microbiota [11,88,89].

The addition of buriti pulp oil (β-carotene: 787.05 mg/kg; α-tocopherol: 689.02 mg/kg, and monounsaturated fatty acids: 91.30 g/100 g) to the diet of Wistar rats (7 g/100 g of diet for 17 days) reduced oxidative damage induced by oral iron overload (FeSO_4_, 60 mg/kg). Buriti oil ingestion reduced LDL-c and hemoglobin and increased monocytes and superoxide dismutase (SOD) activity in serum and liver, thereby reducing oxidative damage [120].

### 4.5. Guava (Psidium guajava L.)

Guava is a tropical fruit of America, widely distributed in tropical and subtropical regions worldwide, and largely produced and consumed in Brazil. The stimulatory effects of guava by-product (20%, *w*/*v*), either submitted or not submitted to a simulated GID, on *L. acidophilus* LA-05, *L. casei* L-26, and *B. lactis* BB-12 were investigated. Guava by-product increased the viable cell counts of the tested probiotics during 48 h of cultivation, regardless of the previous exposure to simulated GID. Tested probiotic strains used guava by-product to support growth and metabolism, increasing the production of organic acids, namely citric, succinic, lactic, acetic, formic, and malic acids, and sugar consumption, besides decreasing the pH value in the cultivation media over time. These results indicate that guava by-product could be a fermentable substrate for probiotic strains, inducing the production of beneficial metabolites [16].

In the same study, the in vitro colonic fermentation of pre-digested guava by-product increased the relative abundance of *Bifidobacterium* spp. (from 2.30% to 4.90%), *Lactobacillus* spp./*Enterococcus* spp. (3.20% to 5.50%) and *E. rectale*/*C. coccoides* (from 4.3% to 4.8%), decreased the relative abundance of *Bacteroides* spp./*Prevotella* spp. (from 4.6% to 4.1%), and increased the contents of acetic, butyric, and propionic acids over time, which were considered indicative of beneficial modulation of the composition and metabolic activity of the intestinal microbiota [16].

The supplementation of guava by-product (400 mg/kg for 28 days) in diet-induced dyslipidemic Wistar rats reduced food intake and body weight, increased the fecal fat excretion, and decreased fat absorption. Total cholesterol, triglycerides, LDL-c, and VLDL levels decreased in animals supplemented with guava by-product. Additionally, the fecal pH and viable cell counts of Enterobacteriaceae were reduced, while the counts of *Bifidobacterium* spp. and *Lactobacillus* spp. were increased in animals supplemented with guava by-product. The animals supplemented with guava by-product kept the integrity of the crypts, goblet cells, and epithelial cells of the intestine, indicating improved colon health. The study also showed that guava by-product has high insoluble fiber content, and phenolic compounds of 3,4-dihydroxybenzoic acid, myricetin, and salicylic acid [15], which could mostly account for the intestinal microbiota modulation, and reduction in fat absorption, accumulation, and metabolization.

Another study investigated guava-leaf aqueous extract (7.0 g/kg/d, 12 weeks) to treat db/db (diabetes and obesity preclinical model) mice. Guava-leaf extract supplementation reduced hyperglycemia, enhanced glucose tolerance and insulin sensitivity, and decreased inflammatory cell infiltration and fibrosis. The guava-leaf extract reduced creatinine (an indicator of renal impairment), ALT (alanine aminotransferase), and total cholesterol plasma levels, besides improving insulin sensitivity in hepatocytes and reducing the expression of G6pc (one of the key enzymes in gluconeogenesis and glycogenolysis). The synergistic action of multiple bioactive components in guava extract could contribute to several clinical outcomes resulting in anti-diabetic effects [33]. The results from 16S rRNA fecal microbiota assessment showed that the treatment with guava-leaf aqueous extract enhanced bacterial richness with an increased relative abundance of *Bacteroidetes*, decreased relative abundance of *Firmicutes* and *Actinobacteria*, and decreased *Firmicutes* to *Bacteroidetes* ratio. At the family level, guava by-product increased the relative abundance of Muribaculaceae, Lachnospiraceae, Lachnospiraceae_ NK4A136_group, and Ruminococcaceae in feces. These microorganisms are associated with improvements in metabolic disorders, including DM [33,59,121], indicating beneficial microbiota alteration in mice treated with guava-leaf extract. The health benefits of guava leaves have been linked to a variety of phenolic compounds, including quercetin, avicularin, apigenin, kaempferol, hyperin, myricetin, gallic acid, catechin, epicatechin, chlorogenic acid, epigallocatechin gallate, and caffeic acid [109].

Guava polysaccharide, i.e., an extract composed of galacturonic acid, galactose, and arabinose from guava, was investigated for its capability of reverting the effects of an HFD in C57BL/6 mice (100 mg/kg for 7 weeks). Guava polysaccharide administration ameliorated body weight gain (decrease of approximately 50%), decreased energy intake, reduced triglycerides, total cholesterol, LDL-c, blood glucose, ALT, and aspartate transaminase (AST) levels, improved glucose intolerance and insulin sensitivity, and decreased lipid accumulation in the liver tissue and hepatic TNF-α and NF-κB levels [56]. A previous study showed that polysaccharides, such as L-arabinose, slow down the absorption of sucrose-derived glucose, which could contribute to the reported effects of guava polysaccharides [122]. In the same study, the bacterial communities in fecal samples were assessed using 16S rRNA sequencing. Supplementation with guava polysaccharide decreased the *Firmicutes* to *Bacteroidetes* ratio (from 4.11 to 1.57%), promoted the growth of beneficial bacteria, i.e., *Enterorhabdus* (0.12% to 0.39%), and increased the contents of SCFA, especially of butyric acid (1.5-fold increase). The same protocol was performed with continuous antibiotic exposure to investigate the mediation of the intestinal microbiota in the physiological effects of guava polysaccharides. The reduction in the intestinal microbiota caused by antibiotic exposure decreased the positive effects induced by guava polysaccharide, indicating the contribution of the composition and metabolic activity of this microbial consortium to reach the extra-intestinal outcomes [56].

The therapeutic effects of a guava aqueous extract (0.05 mL/10 g for 5 days) were investigated in mice with diarrhea induced by *Folium Sennae* (0.05 mL/10 g for at least 9 days). The guava extract administration effectively inhibited diarrhea in mice and increased intestinal microbiota α-diversity. A greater reduction in the relative abundance of *Bacteroidetes* (25%) was found in mice with diarrhea, while a smaller reduction (19%) was found in mice receiving the guava extract. The relative abundance of *Deferribacteraceae* was reduced in mice receiving guava extract [108], suggesting that the hydrophilic compounds from guava could restore diarrhea outcomes in intestinal microbiota.

### 4.6. Juçara (Euterpe edulis Mart.)

Juçara is a native tree of the Atlantic Rainforest found mainly in the southern and southeastern regions of Brazil. The juçara pulp exposed to a simulated GID and further to 24 h of colonic fermentation increased the relative abundance of *Bifidobacterium* spp. (8.5 ± 0.7 log) and Clostridial cluster IX (7.50 ± 0.25 log) populations rather than FOS (log 7.6 ± 0.09 and log 6.59 ± 0.07, respectively). The relative abundance of domain bacteria increased (from 8.32 ± 0.26 to 8.96 ± 0.39 log), while the relative abundance of *C. histolyticum* decreased to below the detection limit. Juçara pulp colonic fermentation produced contents of propionic and acetic acids like FOS after 24 h [91].

The phenolic compounds detected in juçara pulp were cyanidin-3-rutinoside, cyanidin-3-glucoside, malvidin-3-glucoside, peonidin-3-rutinoside, pelargonidin-3-glucoside, rutin, quercetin, and p-coumaric acid. The exposure of juçara pulp to simulated GID decreased the contents of anthocyanins and flavonols and increased p-coumaric acid. Colonic fermentation increased the contents of benzoic acid, followed by gallic and syringic acids [91]. Previous studies indicated that non-absorbable phenolic compounds could be selectively metabolized by the intestinal microbiota and stimulate the growth of beneficial bacterial groups, leading to increased production of metabolites or hydrolyzed compounds more absorbable and/or capable of exerting positive outcomes in health via gut–brain axis modulation [19,81].

A previous study assessed the effects of the supplementation with freeze-dried juçara (0.5%) of an HFD in C57Bl/6 mice for 16 weeks. Juçara reduced and retarded body weight gain, increased energy expenditure, improved glucose tolerance, and reduced fat liver accumulation. These outcomes were associated with the phenolic compounds found in juçara, especially flavonoids and anthocyanins, which were inversely related to body weight gain [123]. Additionally, browning white adipose tissue was linked to juçara ingestion and improvements in energy expenditure [124].

A double-blind, randomized controlled trial with young adults (31 to 59 years) with obesity [body mass index (BMI) ≥ 30 ≤ 39.9 kg/m^2^] tested the consumption of freeze-dried juçara pulp (5 g for 42 days) and reported no alteration in BMI or LPS serum concentrations. However, the consumption of freeze-dried juçara pulp increased the relative abundance of the fecal populations of *A. muciniphila* (an increase of 239.6%), *Bifidobacterium* spp. (an increase of 182.6%), and *C. coccoides* (an increase of 214%). The increase in the relative abundance of *Bifidobacterium* spp. was positively correlated with SCFA concentration in the feces, especially acetic acid [10].

### 4.7. Jabuticaba (Myrciaria jaboticaba (Vell.) Berg)

Jabuticaba, a Brazilian berry in the Atlantic forest, is a highly perishable fruit with dark peels rich in phenolic compounds. A dense, dark-colored by-product, composed of seeds and peels, is generated during industrial jabuticaba processing. The alterations in the relative abundance of different microbial populations during 48 h of in vitro colonic fermentation of jabuticaba by-product submitted to simulated GID were investigated. The colonic fermentation of jabuticaba by-product increased the relative abundance of *Lactobacillus* spp./*Enterococcus* spp. (from 4.32 to 6.25%), *Bifidobacterium* spp. (from 4.60–10.03%), and *Bacteroides* spp./*Prevotella* spp. (from 7.50 to 10.71%), and reduced the relative abundance of *E. rectale*/*C. coccoides* (from 1.97 to 1.37%) and *C. histolyticum* (from 1.32 to 0.91%). Furthermore, the contents of acetic (0.70–1.30 g/L), butyric (1.85–2.30 g/L), and propionic acid (0.68–1.07 g/L) increased, pH values decreased (6.81–4.35), and glucose (from 0.82 to <0.02 g/L) and fructose content (from 0.91 to <0.04 g/L) decreased during colonic fermentation, indicating intense metabolization of jabuticaba by-product by intestinal microbiota [17].

The exposure of jabuticaba by-product to simulated GID resulted in reduced contents of nine phenolic compounds: caftaric acid, gallic acid, cyanidin 3-glucoside, delphinidin 3-glucoside, procyanidin A2, procyanidin B2, hesperidin, *cis*-resveratrol, and rutin. In turn, the contents of epigallocatechin gallate and epicatechin gallate increased after exposure to the simulated GID. The variation in phenolic compound concentration could be due to the diverse responses to pH changes and the influence of digestive enzymes and bile acids, leading to the release and/or degradation of phenolic compounds from fruit material. However, the variety of phenolic compounds reaching the colon could be metabolized by gut microbiota [17].

Peel and seeds of jabuticaba (5%, 10%, and 15%, *w*/*w*) were incorporated into an HFD (50% of fat) typically used to induce obesity in C57BL/6 J mice for 13 weeks. HFD supplemented with jabuticaba peel and seeds (JPS) had ellagic (50%) and gallic acid (46%) as the most abundant phenolic compounds, while cyanidin-3-*O*-glucoside and delphinidin-3-*O*-glucoside were the most abundant anthocyanins (0.7%). The urinary excretion of ellagic acid metabolites by mice receiving an HFD with 10 and 15% of JPS was approximately three-fold higher than those receiving an HFD with 5% of JPS. The ingestion of JPS negatively correlated with weight gain. An HFD with 10 and 15% of JPS improved glucose sensitivity (reduction of 43 and 47%, respectively) and insulin, IL-6 (interleukin-6), and TNF-α plasma levels, besides decreasing aspartate aminotransferase (AST) and ALT levels (by up to 21 and 35 U/L, respectively), liver lipid accumulation (around 60% of reduction), liver fibrosis, tissue inflammation, and expression of hepatic lipid metabolism genes (HMGCoA, SREBP-1, and AMPK). JPS restored mucin 2 and claudin 1 linked to improved intestinal barrier and reduced LPS concentrations in plasma. 16S rRNA assay showed that fecal samples of mice receiving JPS (10 and 15%) reverted changes in the microbial community associated with HFD, including increasing the relative abundance of Desulfovibrionaceae, Clostridiaceae, Peptostreptococcaceae, and Anaeroplasma and decreasing the relative abundance of *Firmicutes* to *Bacteroidetes* ratio (3-folds) [29].

The administration of a hyperlipidic (35% of fat) or normolipidic diet supplemented with freeze-dried jabuticaba peel (4% *w*/*w*) containing gallic acid (11.39 ± 1.58 mg/100 g), cyanidin-3-*O*-glucoside (675.74 ± 51.57 mg/100 g), delphinidin-3-*O*-glucoside (63.67 ± 2.67 mg/100 g), malvidin-3-*O*-glucoside (17.27 ± 1.57 mg/100 g), pelargonidin-3-*O*-glucoside (10.3 ± 0.17 mg/100 g), and peonidin (2.79 ± 0.8 mg/100 g) for thirteen weeks improved insulin sensitivity, reduced fasting glucose, and increased cholesterol excretion in mice fed a hyperlipidic diet. Improvements in cardiometabolic parameters after jabuticaba peel supplementation were accompanied by beneficial changes in the intestinal microbiota linked to the decreased relative abundance of *Firmicutes* and Actinobacteriota and increased relative abundance of Muribaculaceae and Lachnospiraceae family, as well as the changes in *Faecalicatena* genus. *Bifidobacterium* spp., *Lactobacillus* spp., and Atopobiaceae family negatively correlated with feed intake and total cholesterol. In contrast, *Laawsonibacter* spp., *Duncaniella* spp., *Faecalicatena* spp., *Dorea* spp., and Muribaculae family correlated negatively with glycemia and butyric and propionic acid contents. In general, twenty-two measured bacteria genera correlated with some measured parameters (e.g., weight gain, adipose tissue, feed intake, feed efficiency, glycemic cholesterol, butyric acid, and propionic acid), suggesting the role of the intestinal microbiota in the clinical conditions [55].

An aqueous extract of jabuticaba peel, containing the phenolic compounds cyanidin-3-*O*-glucoside, delphinidin-3-*O*-glucoside, gallic acid, rutin, myricetin, and quercetin, was tested (141.1 to 151.4 mg GAE/kg) in Wistar rats with induced colitis at six and seven weeks after colitis induction. Jabuticaba peel extract (215.1 to 208.0 ± 9.7 mg GAE/kg) was tested from the second week until the seventh week after colitis induction. Rats receiving the jabuticaba peel extract had lower weight loss, splenomegaly attenuation, and increased viable cell counts of *Lactobacillus* spp. (short treatment: 7.8 CFU/g; long treatment: 8.0 CFU/g) and *Bifidobacterium* spp. (short treatment: 7.8 CFU/g; long treatment: 8.1 CFU/g) in cecal content. Total SCFA and acetic and butyric acid contents increased in animals receiving jabuticaba peel extract, while IL-6 and colonic TNF-α reduced. Histological investigation indicated that jabuticaba peel extract improved intestinal barrier function by preserving crypts and glands lined by goblet and absorptive cells, submucosa/muscularis mucosae, and muscular layer thickness. The results indicated that jabuticaba peel extract reduced the severity of colitis by stimulating beneficial microbiota composition and metabolism [79].

A previous Investigation evaluated the effects of consuming yogurt (10 mL/kg) supplemented with jabuticaba seed extract (4%, *w*/*v*) in Wistar rats with induced colon cancer for 54 days. The rats receiving yogurt had an increased relative abundance of the phylum *Firmicutes* (58.33%) in the intestinal microbiota. The jabuticaba seed extract had high total phenolic content (57.16 g/100 g), where ellagitannins (10.1 g/100 g), ellagic acid (98%), proanthocyanidins (3.00 g/100 g), and prodelphinidins (63%) are the most prevalent phenolic compounds [106]. The yogurt supplemented with jabuticaba peel extract protected DNA strand scission and LDL oxidation against DNA damage (inhibiting 90.4 ± 3.7% of the DNA scission), and decreased total aberrant crypt foci (87.8 ± 38.9 to 123.8 ± 47.8) and expression of COX-2 (cyclooxygenase-2) linked to reduced inflammation. Consumption of yogurt supplemented with jabuticaba seed extract increased Bacteroidetes phylum and equalized the abundance of total bacterial and *Gammaproteobacteria*, which were microbial alterations in rats with induced cancer. The high content of phenolic compounds, especially the ellagitannins, was putatively associated with inflammation reduction and microbiota modulation, which improved cell apoptosis and colon cancer progression [106].

A yogurt supplemented with freeze-dried jabuticaba seed extract (53.94 g/100 g GAE), containing 39% of total phenolic compounds including castalagin, vescalagin, procyanidin A2, and ellagic acid, was tested in Wistar rats with 1,2-dimethylhydrazine induced-colon cancer. Consuming yogurt supplemented with lyophilized jabuticaba seed extract increased alpha diversity and equalized bacterial biodiversity in feces. It also promoted cytotoxic effects on cancer cells and exhibited antioxidant activity by reducing reactive oxygen species generation [90].

### 4.8. Passion Fruit (Passiflora capsularis L.)

Passion fruit is found in tropical and semitropical regions of the world. Brazil is the major passion fruit producer, remarkably in the Brazilian northeast region. Cow-fermented milk supplemented with passion fruit pulp (1%, *w*/*v*) was evaluated for its capability to modulate the intestinal microbiota of healthy individuals (18–22 years old). The milk was fermented using *L. casei* and *L. plantarum* before supplementing with passion fruit pulp, and further administrated for 5 days to SHIME^®^ fermentation. The microbiota composition was evaluated using 16S rRNA sequencing. Fermented milk supplemented with passion fruit pulp had high viable cell counts of *L. casei* (9.59 Log CFU/mL) and *L. plantarum* (9.43 Log CFU/mL). It also increased the relative abundance of commensal microorganisms, i.e., *Phocaeicola* and *Mediterraneibacter* genera, in the intestinal microbiota, which were predominant at the end of the SHIME^®^ fermentation. In addition, fermented milk supplemented with passion fruit pulp increased the content of acetic (52.65–125.66 mmol/L) and butyric acid (7.72–15.96 mmol/L) during SHIME^®^ fermentation [48].

The effect of low-fat fermented goat milk supplemented with passion fruit by-product (17.96 mg GAE/100 g) on the human intestinal microbiota was evaluated using SHIME^®^, pooled stool samples from three adult volunteers with obesity (body mass index between 30 and 39.9 Kg/m^2^ and waist circumference > 80 cm), 16S rRNA sequencing, and SCFA quantification [44]. The fermented goat milk supplemented with passion fruit by-product increased the relative abundance of *Sutterella*, *Klebsiella*, *Collinsella*, *Dialister*, *Lactobacillus*, and *Bidifobacterium* genera during the first and second week of fermentation in SHIME^®^, besides reducing the relative abundance of bacteria belonging to *Prevotella*, *Megamonas*, and *Succinivibrio* genera in the simulated colonic region. *Collinsella*, *Coraliomargarita*, *Desulfovibrio*, *Elusimicrobium*, *Holdemanella*, *Parabacteroides*, *Pseudoflavonifractor*, and *Subdogranilum* positively correlated with butyric acid content [44]. Passion fruit by-product is reported as a rich source of insoluble and soluble dietary fiber, besides having high contents of total phenolic compounds [93], which could drive its beneficial effects on intestinal microbiota.

The administration of soluble dietary fiber from yellow passion fruit (100 mg/kg) to Swiss mice with dextran sodium sulfate (DSS)-induced colitis reduced body weight loss, ameliorated colon length reduction, restored glutathione (GSH) and SOD activity, reduced IL-1β and TNF-α levels, and increased IL-10 levels. In the same study, soluble dietary fiber from passion fruit reduced histopathological damage and improved intestinal colon mucus layer impaired by colitis [125]. Soluble fibers could enhance the production of metabolites from intestinal microbiota and reduce the transcription of proinflammatory mediators, thereby reducing inflammatory diseases [4,125]. The pathogenesis of ulcerative colitis is not understood; however, the intestinal epithelium damage associated with the dissemination of proinflammatory luminal mediators by microbial antigens has been described as an essential factor in ulcerative colitis progression, showing a potential role of the intestinal microbiota in its development. It suggests that bioactive compounds targeting the intestinal microbiota could produce beneficial outcomes in ulcerative colitis [79,125].

Altogether, the evidence from available literature has shown that Brazilian native fruits and their by-products could support the growth of probiotic bacteria found as part of the human intestinal microbiota, as well as that colonic fermentation of pulp, peel, and/or seeds of these fruits can selectively stimulate the proliferation of beneficial bacterial intestinal groups, and drive the metabolic activity of intestinal microbiota to produce several metabolites linked to local and systemic health effects in the host. The modulatory impacts of Brazilian native fruits on the composition and metabolic activity of the intestinal microbiota could improve several clinical repercussions associated with NCDs, reinforcing the influence of intestinal microbiota in extra-intestinal outcomes. However, the consumption of Brazilian native fruits to increase the beneficial modulatory effects on the intestinal microbiota must be part of an overall healthy diet pattern, which is high in fresh fruits and vegetables, legumes, seeds, whole grains, and nuts, and low in animal-based foods, mostly fatty and processed meats; it should also be linked to a healthy lifestyle that includes non-smoking, regular exercise, ideal weight, and modest alcohol consumption, as they are the major factors protecting against NCDs [1,2,3,4,5,6].

## 5. Conclusions

Several investigations have shown the role of intestinal microbiota in NCD development and related risk factors due to intestinal and extra-intestinal impairments, including damage to intestinal integrity, increased LPS concentration, inflammation signaling, and increased blood–brain barrier permeability. Brazilian native fruits, such as acerola, açaí, baru, buriti, guava, jabuticaba, juçara, and passion fruit (pulp, peel, and/or seed), and their by-products have several bioactive compounds, such as soluble and insoluble fibers and a variety of phenolic compounds, which are capable of restoring these alterations. Brazilian native fruits and their by-products could exert a regulatory influence on the intestinal microbiota composition, resulting in favorable effects on both intestinal and systemic health. These effects could be associated with the capability of Brazilian native fruits to reduce energy intake and absorption, increase SCFA production, enhance intestinal integrity and homeostasis, and reduce fecal pH. However, further research could focus on better understanding the specific microbial groups involved in metabolite production and their corresponding outcomes in NCD development. Further research should also identify the specific components in Brazilian native fruits for directing the production of microbial metabolites by the intestinal microbiota associated with systemic health benefits affecting NCD development.

## Figures and Tables

**Table 1:** Main bioactive compounds in Brazilian native fruits and their by-products related to intestinal microbiota modulation.

Fruits	Main Bioactive Compounds in Brazilian Native Fruits Related to Intestinal Microbiota Modulation	Reference
Acerola (*Malpighia emarginata* D.C.)		
Pulp	Phenolic compounds: cyanidin3-rhamnoside: 68.22 ± 0.71 mg/100 g; (+)-catechin: 2.50 ± 0.25 mg/100 g; isorhamnetin: 4.53 ± 0.25 mg/100 g.	[9]
By-product	Total dietary fibers: 48.57 ± 0.39 g/100 g; insoluble dietary fibers: 34.35 ± 0.29 g/100 g; soluble dietary fibers: 14.22 ± 0.10 g/100 g; phenolic compounds: 2,5-dihydroxybenzoic acid: 737.20 ± 0.4 mg/100 g; catechin: 125.20 ± 0.09 mg/100 g; myricetin: 94.20 ± 0.01 mg/100 g; salicylic acid: 348.64 ± 0.51 mg/100 g; rutin: 68.60 ± 0.05 mg/100 g.	[15]
Açaí (*Euterpe oleracea* Mart.)		
Whole fruit	Total phenolic compound concentrations: 32.00 ± 1.03 mg GAE/100 g; total flavonoids: 6.39 ± 1.23 mg quercetin equivalent/g; total anthocyanins: 10.20 ± 0.24 mg cyanidin-3-glucoside equivalent/g; total proanthocyanidins: 6.10 ± 2.09 mg procyanidin B1 equivalent/g.	[8]
Baru (*Dipteryx alata* Vog.)		
Pulp	Total dietary fibers: 32.90 ± 0.42 g/100 g; insoluble dietary fibers: 32.90 ± 0.42 g/100 g; soluble dietary fibers: <0.10 g/100 g; fructose: 7.76 ± 0.82 g/100 g; glucose: 6.32 ± 0.70 g/100 g; phenolic compounds: hesperidin: 19.34 ± 0.10; procyanidin B2: 0.53 ± 0.00 mg/100 g; epicatechin gallate: 0.49 ± 0.01 mg/100 g; chlorogenic acid: 0.46 ± 0.01 mg/100 g; catechin: 0.43 ± 0.00 mg/100 g; total phenolic compounds: 507.01 ± 15.41 mg GAE/100 g; total tannins: 142.91 ± 7.16 mg TAE/100 g (trolox equivalent).	[81]
Buriti (*Mauritia flexuosa* L.)		
Fresh pulp	Total dietary fibers: 38.0 ± 0.3 g/100 g; insoluble dietary fibers: 27.3 ± 0.4 g/100 g; soluble dietary fibers: 10.6 ± 0.3 g/100 g.	[88]
Freeze-dried pulp	Total dietary fibers: 38.9 ± 0.6 g/100 g; insoluble dietary fibers: 28.8 ± 1.0 g/100 g; soluble dietary fibers: 10.1 ± 0.2 g/100 g.	
Freeze-dried peel	Total dietary fibers: 50.5 ± 0.6 g/100 g; insoluble dietary fibers: 50.0 ± 1.0 g/100 g; soluble dietary fibers: 0.55 ± 0.03 g/100 g.	
Freeze-dried endocarp	Total dietary fibers: 28.14 ± 0.05 g/100 g; insoluble dietary fibers: 24.66 ± 0.01 g/100 g; soluble dietary fibers: 3.48 ± 0.08 g/100 g.	
Fruit	Total pectin: 0.59 ± 0.02 g/100 g; soluble pectin: 0.49 ± 0.01 g/100 g; total phenolic compounds: 110.72 ± 0.26 mg GAE/100 g (gallic acid equivalents).	[11]
Fruit	Isoquercetin: 156.07; ferulic acid: 135.22 mg GAE/100 g; vanillic acid: 46.94 mg GAE/100 g; caffeic acid: 15.88 mg GAE/100 g; quercetin: 14.61 mg GAE/100 g.	[89]
Guava (*Psidium guajava* L.)		
By-product	Total dietary fibers: 44.36 ± 0.23 g/100 g; insoluble dietary fibers: 40.62 ± 0.24 g/100 g; soluble dietary fibers: 7.98 ± 0.20 g/100 g; phenolic compounds: 3,4-dihydroxybenzoic acid 97.80 ± 0.11 mg/100 g; salicylic acid: 310.60 ± 0.89 mg/100 g; 2,5-dihidorxybenzoic acid: 89.40 ± 0.78 mg/100 g; myricetin: 44.60 ± 0.01 mg/100 g; synapic acid: 18.80 ± 0.0 mg/100 g.	[15]
Jaboticaba (*Myrciaria jaboticaba* (Vell.) Berg)		
Seed	Total phenolic compound concentrations: 53,944 ± 773 mg GAE/100 g; phenolic compounds: castalagin: 13,349 ± 1494 mg/100 g; vescalagin: 6875 ± 1240 mg/100 g; procyanidin A2: 451 ± 81 mg/100 g; ellagic acid: 388 ± 49 mg/100 g; gallic acid: 230 ± 14 mg/100 g.	[90]
Freeze-dried peel	Total dietary fiber: 33.77 ± 1.20 g/100 g; insoluble dietary fiber: 25.34 ± 0.33 g/100 g; soluble dietary fiber (g/100 g): 8.49 ± 0.48 g/100 g; total phenolic compounds: 8219.94 ± 99.56 mg GAE/100 g; phenolic compounds: gallic acid: 11.39 ± 1.58 mg/100 g; cyanidin-3-*O*-glucoside: 675.74 ± 51.57 mg/100 g; delphinidin- 3-*O*-glucoside: 63.67 ± 2.67 mg/100 g; malvidin-3-*O*-glucoside: 17.27 ± 1.57 mg/100 g; pelargonidin-3-*O*-glucoside: 10.3 ± 0.17 mg/100 g; peonidin: 2.79 ± 0.84 mg/100 g.	[55]
By-product	Total dietary fiber: 84.75 ± 1.22 g/100 g; insoluble dietary fiber: 28.49 ± 0.82 g/100 g; soluble dietary fiber: 56.26 ± 1.42 g/100 g; total dietary fiber: 84.75 ± 1.22 g/100 g; fructose: 0.45 ± 0.08 g/100 g; glucose: 0.38 ± 0.05 g/100 g; Total phenolic compounds: 14.44 ± 0.152 mg/100 g; hesperidin: 12.07 ± 0.18 mg/100 g; gallic acid: 2.80 ± 0.05 mg/100 g.	[17]
Juçara (*Euterpe edulis* Mart.)		
Freeze-dried pulp	Phenolic profile: cyanidin-3-rutinoside, cyanidin-3-glucoside, malvidin-3-glucoside, peonidin-3-rutinoside, pelargonidin-3-glucoside, rutin, quercetin, and p-coumaric acid (no quantification of a specific phenolic compound); total phenolic compound concentrations: 3474 ± 98.0 mg.	[91]
Pomace	Monomeric anthocyanins: 7230.0 mg cyanidin 3-*O*-glycoside/100 g; total dietary fibers: 72.7 g/100 g; insoluble dietary fiber: 68.62 ± 0.23 g/100 g; total phenolic compounds: 1380.0 ± 170.0 mg GAE/100 g.	[92]
Passion (*Passiflora capsularis* L.)		
By-product	Total dietary fiber: 64.20 ± 0.28 g/100 g; insoluble dietary fiber: 44.80 ± 0.14 g/100 g; soluble dietary fiber: 19.40 ± 0.14 g/100 g; total phenolic compounds: 384.44 ± 22.50 mg GAE/100 g.	[93]

**Table 2:** Retrieved studies assessing the effects of Brazilian native fruit and their by-products on human intestinal microbiota.

Type of Study	Intestinal Microbiota Condition	Fruit Evaluated	Dose	Reference
In vitro studies				
In vitro: simulated digestion and fluorescence in situ hybridization (FISH)	Healthy volunteers	Acerola by-product (*M. emarginata* D.C.)	ND	[107]
Simulated digestion and fluorescence in situ hybridization (FISH)	Healthy volunteers	Açai fruit pulp (*Euterpe oleracea*).	ND	[87]
The Simulator of the Human Intestinal Microbial Ecosystem (SHIME^®^)	Healthy male volunteers	Milk fermented by *Lacticaseibacillus casei* SJRP38 (2%), *Lactiplantibacillus plantarum* ST8ShStr (2%), and *Streptococcus thermophilus* TA 080 (0.1%) with 1% (*w*/*v*) of passion fruit (*Passiflora edulis* F.) or buriti fruit (*Mauritia flexuosa*) pulp	80 g for 5 days	[48]
The Simulator of the Human Intestinal Microbial Ecosystem (SHIME^®^)	Obese volunteer (body mass index between 30 and 39.9 kg/m^2^ and waist circumference > 80 cm)	Low-fat goat milk fermented by *L. casei* Lc-1 and *S. thermophilus* TA040 with passion fruit by-product (1%, *w*/*v*, seeds, peels and pomace)	400 mL	[44]
Simulated digestion and fluorescence in situ hybridization (FISH)	Healthy volunteers	Freeze-dried juçara fruit (*Euterpe edulis*) pulp	1%	[91]
Simulated digestion and fluorescence in situ hybridization (FISH)	Healthy volunteers	Jabuticaba (*Myrciaria jaboticaba*) by-product	20%	[17]
Simulated digestion and fluorescence in situ hybridization (FISH)	Healthy volunteers	Guava (*Psidium guajava* L.) and acerola (*Malpighia glabra* L.) by-products	20%	[16]
In vitro: Cell-based cytotoxicity evaluation, erythrocyte protection In vivo: Preclinical study	1,2 dimethyl hydrazine-induced colon carcinogenesis in male Wistar rats	Yogurt with (0.4%) or without jabuticaba (*Myrciaria jaboticaba*) seed extract.	10 mL/kg	[90]
Preclinical studies				
Animal model	Dyslipidaemic diet in female Wistar rats	Guava (*Psidium guajava* L.) by-product	800 mg/kg/day for 28 days	[15]
Animal model	Male db/db mice (diabetes and obesity)	Aqueous extract of guava (*Psidium guajava* L.) leaf	7.0 g/kg for 12 weeks	[33]
Animal model	2,4,6-trinitrobenzene sulfonic acid-induced colitis in Wistar rats	Extract of jabuticaba (*Myrciaria jaboticaba*) peel Extract was offered instead of water	From 141.1 ± 16.0 to 215.1 ± 31 mg GAE/kg	[79]
Animal model	1,2-dimethyl hydrazine-inducedcolon carcinogenesis in Wistar rats	Yogurt with (0.4%) or without jabuticaba (*Myrciaria jaboticaba*) seed extract	10 mL/kg	[106]
Animal model	Male C57BL/6 mice	Freeze-dried jabuticaba (*Plinia cauliflora* (Mart.) Kausel) peel	4% (3.28 g/kg of experimental diet)	[55]
Animal model	C57BL/6 mice	Aqueous polysaccharides guava (*Psidium guajava*) extract	100 mg/kg for 11 weeks	[56]
Animal model	Mice diarrhea induced by *Folium Sennae*	Aqueous *Psidium guajava* extract	0.05 mL/10 g	[108]
Animal model	Male C57BL/6 mice	Jabuticaba (*Myrciaria jaboticaba*) seed and peel	5%, 10%, and 15% of diet	[29]
Animal model	Male C57BL/6J mice	Anthocyanin-rich extract of açaí (*Euterpe oleracea*)	150 mg/kg for 4 weeks	[54]
Clinical study				
Double-blind randomized controlled trial	Adults (31 to 59 years) with obesity [body mass index (BMI) ≥ 30 ≤ 39.9 kg/m^2^]	Freeze-dried juçara fruit pulp (*Euterpe edulis*)	5 g for 42 days	[10]

## Data Availability

No new data were created or analyzed in this study. Data sharing is not applicable to this article.
